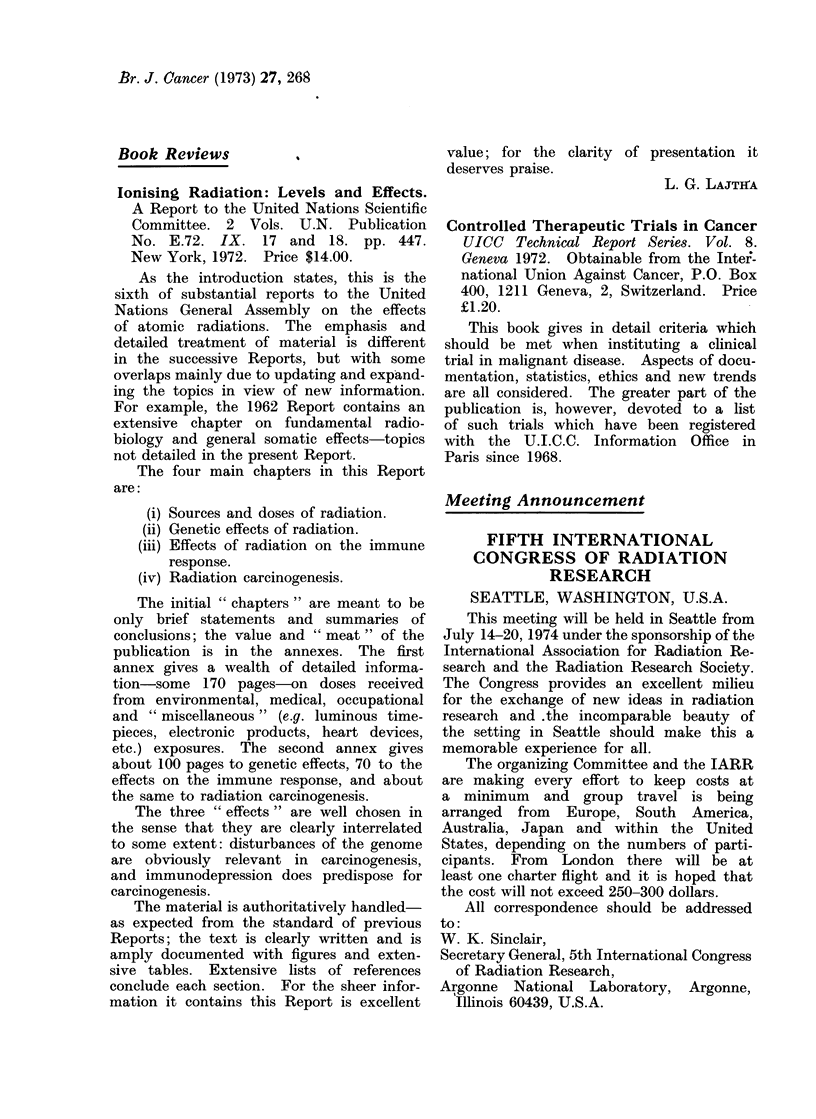# Ionising Radiation: Levels and Effects

**Published:** 1973-03

**Authors:** L. G. Lajtha


Br. J. Cancer (1973) 27, 268

### Book Reviews

**Ionising Radiation: Levels and Effects.** A Report to the United Nations Scientific Committee. 2 Vols. U.N. Publication No. E.72. *IX*. 17 and 18. pp. 447. New York, 1972. Price $14.00.

As the introduction states, this is the sixth of substantial reports to the United Nations General Assembly on the effects of atomic radiations. The emphasis and detailed treatment of material is different in the successive Reports, but with some overlaps mainly due to updating and expanding the topics in view of new information. For example, the 1962 Report contains an extensive chapter on fundamental radiobiology and general somatic effects—topics not detailed in the present Report.

The four main chapters in this Report are:

(i) Sources and doses of radiation.
(ii) Genetic effects of radiation.
(iii) Effects of radiation on the immune response.
(iv) Radiation carcinogenesis.

The initial " chapters " are meant to be only brief statements and summaries of conclusions; the value and " meat " of the publication is in the annexes. The first annex gives a wealth of detailed information—some 170 pages—on doses received from environmental, medical, occupational and " miscellaneous " (*e.g.* luminous timepieces, electronic products, heart devices, etc.) exposures. The second annex gives about 100 pages to genetic effects, 70 to the effects on the immune response, and about the same to radiation carcinogenesis.

The three " effects " are well chosen in the sense that they are clearly interrelated to some extent: disturbances of the genome are obviously relevant in carcinogenesis, and immunodepression does predispose for carcinogenesis.

The material is authoritatively handled—as expected from the standard of previous Reports; the text is clearly written and is amply documented with figures and extensive tables. Extensive lists of references conclude each section. For the sheer information it contains this Report is excellent value; for the clarity of presentation it deserves praise.

L. G. LAJTHA